# Small bowel hemangiomas: Diagnostic role of capsule endoscopy

**DOI:** 10.4103/0971-9261.71755

**Published:** 2010

**Authors:** Sanat Khanna, Ravi P. Kanojia, Prema Menon, Surinder Rana, B. R. Thapa, D. K. Bhasin, K. L. N. Rao

**Affiliations:** Department of Pediatric Surgery, Advanced Pediatric Center, Post Graduate Institute of Medical Education and Research, Chandigarh, India; 1 Department of Pediatric Gastroenterology, Post Graduate Institute of Medical Education and Research, Chandigarh, India

**Keywords:** Capsule endoscopy, intestinal hemangiomas, malena, severe anemia

## Abstract

Vascular anomalies involving the small bowel are an uncommon cause of gastrointestinal bleeding in childhood. We present here an 11-year-old boy who presented with severe anemia and malena. The routine investigations did not reveal any pathology. A capsule endoscopy study was performed, which clinched the diagnosis and identified two intestinal hemangiomas. The hemangiomas were resected and the child recovered.

## INTRODUCTION

Lower gastrointestinal (GI) bleeding in children is most often caused by Meckel’s diverticulum, intussusception, or necrotizing enterocolitis.[1] Vascular anomalies are rare and do not represent a frequent cause of GI bleeding. However, vascular anomalies may cause massive acute or chronic innocuous bleeding resulting in severe anemia. Delay in diagnosis is common, and intraoperative localization of these intraluminal lesions is usually very difficult. The purpose of this report is to highlight the importance and role of capsule endoscopy in identifying such lesions in which all other modalities of investigations fail.

## CASE REPORT

An 11-year-old boy presented with history of parents noticing progressive pallor in the child with easy fatigability and weakness associated with occasional episodes of malena. There was a history of excision of large cutaneous hemangioma over left scapular region at 3 years of age at a local hospital, during which he was transfused 2 units of whole blood.

The clinical examination revealed a grossly pale child with mild tachycardia and a well-healed scar over the left scapula. The general and systemic exam was within normal limits. An initial workup confirmed severe anemia with hemoglobin of 3.5 g%, hematocrit of 14% with normal platelet counts. His stool for occult blood was positive for guaiac test. He was transfused 2 units of blood, following which he underwent upper GI endoscopy and colonoscopy which were essentially normal.

A barium meal follow through also did not reveal anything abnormal, as did all the investigations, to rule out malabsorption syndrome and celiac disease. As his symptoms persisted, a selective mesenteric computed tomogram (CT) angiography was performed which only suggested a mild thickening of the terminal ileum, with no evidence of any cause of lower GI bleed. A red blood cell tagged technitium^99^ (RBC Tc^99^) scan indicated a small collection of blood present at the terminal ileum with no definite cause and site having been cited.

**Figure 1:**
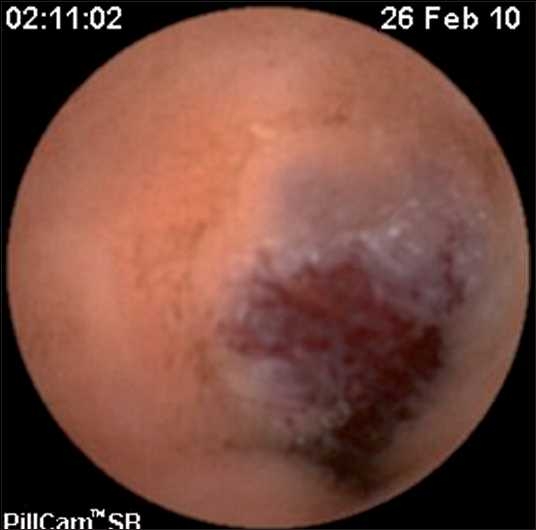
Snapshots of the pictures taken by the capsule looking at the lesion, first lesion

**Figure 2:**
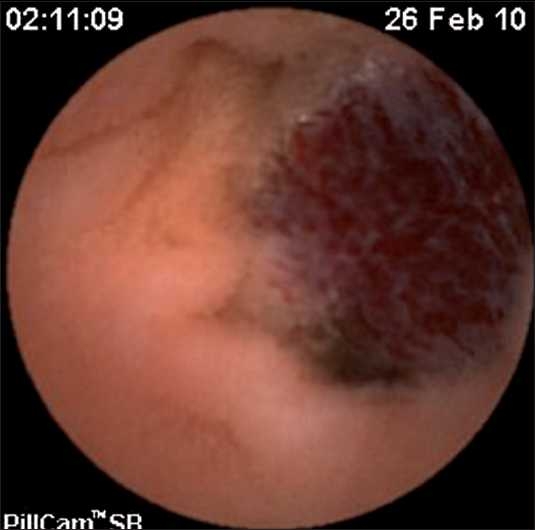
Snapshot of the second lesion

**Figure 3:**
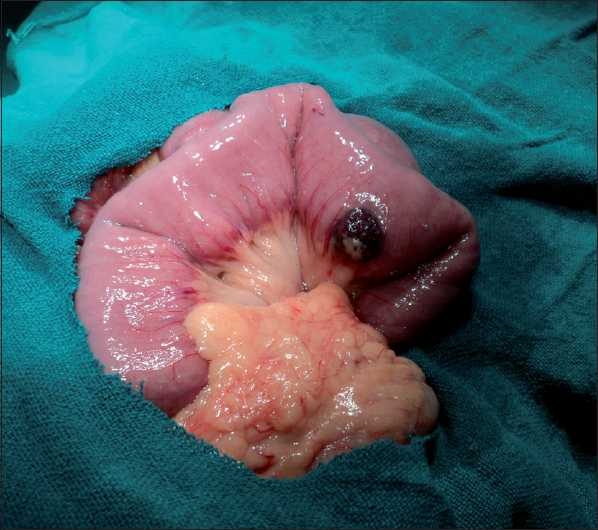
Lesion seen intraoperatively; first lesion is big and obvious and the second lesion is relatively less prominent on the serosal side

**Figure 4:**
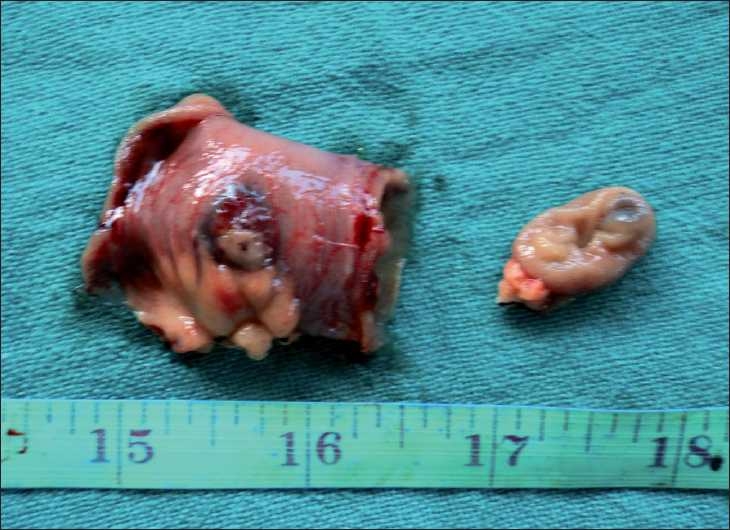
Resected specimen showing the lesion from the luminal side

A capsule endoscopy (using PillCam™) identified two hemangiomatous lesions at the level of mid and terminal parts of jejunum, with evidence of fresh bleed in one of the lesions [Figures 1 and 2]. At laparotomy, there were two lesions seen at mid jejunum and jejuno-ileal junction, approximately 40 cm apart [Figures 3 and 4], involving both the mucosal and serosal aspects of the affected bowel. With the rest of the entire small and large bowel along with solid organs being normal, a resection anastamosis was done at two sites. Postoperatively, the child recovered well and has been thriving at 6 months follow-up.

## DISCUSSION

**Figure 5:**
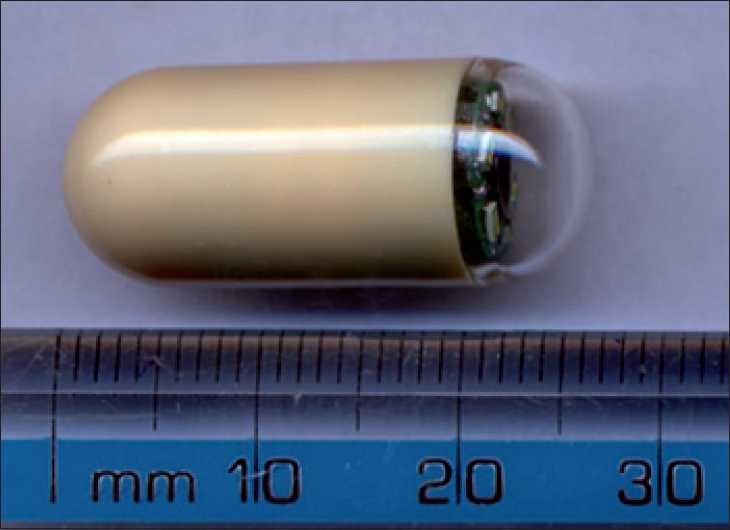
Capsule device used for the endoscopy (note the small size of the capsule making it easy to swallow)

Capsule endoscopy is a technology approved by the Food and Drug Administration, USA, for its use in pediatric age group between 10 and 18 years,[2] and involves swallowing a video capsule of size 26 × 11 mm [Figure 5] to visualize and record its findings in the esophagus, stomach, and small intestine. The patient is required to undergo bowel preparation (as used before colonoscopy) with the use of laxatives, etc. The capsule contains one or two video chips (cameras), a light bulb, a battery, and a radio transmitter.[3]

As the capsule travels through the esophagus, stomach, and small intestine, it takes photographs at the rate of approximately two per second. The photographs are transmitted by the radio transmitter to a small receiver that is worn on the waist of the patient undergoing capsule endoscopy. At the end of the procedure, approximately 24 hours later, about 50,000 photographs are downloaded from the receiver into a computer, and the relevant images are sorted out by the technician, whereas the capsule is passed by the patient into the toilet and flushed away.

Capsule endoscopy has proven to be superior and sensitive in diagnosing lesions which may have been missed on rest of the investigations, and it is especially useful for sites between duodenojejunal (DJ) and ileocecal (IC) junction. One such pathology is angiodysplasia that can bleed intermittently. Literature is abound with case reports of small intestinal lesions such as lymphoma, carcinoid tumor, malignancies and Crohn’s disease of the small intestine, detected and treated using capsule endoscopy, as well as investigation of Peutz-Jeghers syndrome using capsule endoscopy.[4] There have been a few studies to support that the wireless capsule endoscopy indeed increases the yield of various pathologies in pediatric age group.[5] However, some of the limitations of this modality are suspected small bowel strictures, active ongoing heavy bleeding which may obscure all the view, and the age limit (less than 5-6 years) under which its use may require general anesthesia in order to place the capsule into the duodenum using an endoscope.

Intestinal hemangiomas are most commonly found in the mid-jejunum and may be solitary or occasionally multiple. These can present with a spectrum of clinical picture like anemia, which may be innocuous or chronic and severe, malena, abdominal pain, intussusceptions, intramural hematoma and/or intestinal obstruction with or without perforation. The diagnosis of intestinal hemangiomas is difficult and the treatment of these lesions remains primarily surgical; however, intraoperative localization is a big challenge.

Barium studies of the GI tract are not very useful,[6] whereas nuclear medicine scans are more often than not negative. The interpretation of these tests is associated with a significant percentage of false-positives.[7] Upper GI and lower GI endoscopies, although necessary, are of little help in localizing the lesion, whereas selective angiography along with RBC tagged Tc^99^scan are a relatively reliable way to diagnose and determine the location of these lesions, but their sensitivity increases if the patient is actively bleeding and thus their limitations.

To conclude, this case highlights the role of capsule endoscopy in clinching the diagnosis in which other modalities were of little help.

## References

[1] Fremond B, Yazbeck S, Dubois J, Brochu L, Ouimet GA (1997). Intestinal vascular anomalies in children. J Pediatr Surg.

[2] (2003). FDA approves capsule endoscopy for children 10 to 18 years of age. Hayes Alert.

[3] http://www.MedicineNet.com.

[4] Postgate A, Hyer W, Phillips R, Gupta A, Burling D, Bartram C (2009). A Feasibility of Video Capsule Endoscopy in the Management of Children With Peutz-Jeghers Syndrome: A Blinded Comparison With Barium Enterography for the Detection of Small Bowel Polyps. J Paediat Gastroenterol Nutr.

[5] Thomson M, Fritscher-Ravens A, Mylonaki M, Swain P, Eltumi M, Heuschkel R (2007). Wireless capsule endoscopy in children: A study to assess diagnostic yield in small bowel disease in paediatric patients; J Pediatr Gastroenterol Nutr.

[6] Abrahamson J, Shandlmg B (1973). Intestinal hemangiomata in childhood and a syndrome for diagnosis: A collective review. J Pediatr Surg.

[7] Whitaker SC, Perkins AC, Wastie ML (1993). The value of scintigraphic studies m the assessment of patients with acute or chronic gastromtestinal haemorrhage. Nucl Med Com.

